# Assisted mechanical ventilation promotes recovery of diaphragmatic thickness in critically ill patients: a prospective observational study

**DOI:** 10.1186/s13054-020-2761-6

**Published:** 2020-03-12

**Authors:** Alice Grassi, Daniela Ferlicca, Ermes Lupieri, Serena Calcinati, Silvia Francesconi, Vittoria Sala, Valentina Ormas, Elena Chiodaroli, Chiara Abbruzzese, Francesco Curto, Andrea Sanna, Massimo Zambon, Roberto Fumagalli, Giuseppe Foti, Giacomo Bellani

**Affiliations:** 1 School of Medicine and Surgery, University of Milano-Bicocca, Monza, Italy; 2 Department of Anesthesia and Intensive Care Medicine, ASST Monza, Monza, Italy; 3 Department of Pathophysiology and Transplantation, University of Milan, Milan, Italy; 4 Department of Anesthesia, Critical Care and Emergency, Fondazione IRCSS Ca’ Granda, Ospedale Maggiore Policlinico, Milan, Italy; 5 Neurocritical Care Unit, ASST Grande Ospedale Metropolitano Niguarda, Milan, Italy; 6 Department of Anesthesia and Intensive Care Medicine, Cernusco sul Naviglio Hospital, ASST Melegnano e Martesana, Milan, Italy

**Keywords:** Diaphragm ultrasound, Assisted mechanical ventilation, Diaphragm thickness

## Abstract

**Background:**

Diaphragm atrophy and dysfunction are consequences of mechanical ventilation and are determinants of clinical outcomes. We hypothesize that partial preservation of diaphragm function, such as during assisted modes of ventilation, will restore diaphragm thickness. We also aim to correlate the changes in diaphragm thickness and function to outcomes and clinical factors.

**Methods:**

This is a prospective, multicentre, observational study. Patients mechanically ventilated for more than 48 h in controlled mode and eventually switched to assisted ventilation were enrolled. Diaphragm ultrasound and clinical data collection were performed every 48 h until discharge or death. A threshold of 10% was used to define thinning during controlled and recovery of thickness during assisted ventilation. Patients were also classified based on the level of diaphragm activity during assisted ventilation. We evaluated the association between changes in diaphragm thickness and activity and clinical outcomes and data, such as ventilation parameters.

**Results:**

Sixty-two patients ventilated in controlled mode and then switched to the assisted mode of ventilation were enrolled. Diaphragm thickness significantly decreased during controlled ventilation (1.84 ± 0.44 to 1.49 ± 0.37 mm, *p* < 0.001) and was partially restored during assisted ventilation (1.49 ± 0.37 to 1.75 ± 0.43 mm, *p* < 0.001). A diaphragm thinning of more than 10% was associated with longer duration of controlled ventilation (10 [5, 15] versus 5 [4, 8.5] days, *p* = 0.004) and higher PEEP levels (12.6 ± 4 versus 10.4 ± 4 cmH_2_O, *p* = 0.034). An increase in diaphragm thickness of more than 10% during assisted ventilation was not associated with any clinical outcome but with lower respiratory rate (16.7 ± 3.2 versus 19.2 ± 4 bpm, *p* = 0.019) and Rapid Shallow Breathing Index (37 ± 11 versus 44 ± 13, *p* = 0.029) and with higher Pressure Muscle Index (2 [0.5, 3] versus 0.4 [0, 1.9], *p* = 0.024). Change in diaphragm thickness was not related to diaphragm function expressed as diaphragm thickening fraction.

**Conclusion:**

Mode of ventilation affects diaphragm thickness, and preservation of diaphragmatic contraction, as during assisted modes, can partially reverse the muscle atrophy process. Avoiding a strenuous inspiratory work, as measured by Rapid Shallow Breathing Index and Pressure Muscle Index, may help diaphragm thickness restoration.

## Introduction

Diaphragm dysfunction during mechanical ventilation is increasingly recognized as a clinical entity which has an impact on important clinical outcomes, such as the risk of prolonged mechanical ventilation, re-intubation, tracheostomy, and death [1]. Ultrasound (US) is becoming a reference to assess diaphragmatic injury and to detect changes in diaphragmatic thickness over time; both an increase and a decrease of diaphragmatic thickness are associated with diaphragm activity and ventilation outcome [1, 2]. Starting from experimental and clinical studies, four mechanisms of diaphragmatic injury (myotrauma) have been identified: disuse atrophy due to over-assistance [3], excessive load due to under-assistance [1, 4], eccentric myotrauma from diaphragm contraction during expiration [5] or asynchronies, and longitudinal atrophy, due to high level of positive end-expiratory pressure (PEEP) [6]. Therefore, the need to set “protective” mechanical ventilation not only for the lungs but also for the diaphragm was recently advocated [7]. The targets of this type of ventilation would be to avoid excessive or minimal diaphragm activity, trying to mimic the inspiratory effort of healthy subjects [8]. In fact, a diaphragmatic thickening fraction between 25 and 40% (considered the reference range for resting tidal breathing) was associated with stable diaphragmatic thickness [2], shorter duration of mechanical ventilation [1], and higher incidence of weaning success [9]. Given all of these, it is advisable to strictly monitor patients’ inspiratory efforts, and specifically diaphragmatic function, while avoiding lung injury [10].

Some clinical studies showed that intermittent diaphragm stimulation during cardiothoracic surgery could exert benefits on diaphragmatic force generation and mitochondrial function [11, 12]. On the other side, experimental studies showed that the mode of ventilation could influence the degree of diaphragmatic dysfunction. In healthy rats, controlled mechanical ventilation (CMV) lead to proteolysis and decreased protein synthesis, while the same period spent under pressure support ventilation (PSV) preserved diaphragmatic protein content, with no increase in oxidative injury [13]. In another animal study, assisted mechanical ventilation (AMV) prevented atrophy and loss of diaphragmatic force caused by controlled ventilation [14]. Recently, the first study conducted on humans (two groups of organ donors as study groups and thoracic surgery patients as controls) was published showing a correlation between the severity of diaphragm histological damage and the mode of ventilation used. In particular, the duration of the period without diaphragm stimuli (i.e. CMV) correlated with a decreased cross-sectional area of the fibres in both study groups compared to controls [15]. One of the two study groups underwent a longer period of CMV than the other organ donors group, but this did not lead to a significant reduction in diaphragm fibre cross-sectional area. The same group spent more time also under an assisted mode of ventilation, with some preservation of diaphragmatic function, and this was advocated as the possible mechanism which avoided excessive atrophy.

The aim of this study is to test the hypothesis that, in the face of decreased diaphragmatic thickness during CMV, assisted modes of ventilation which partially preserves diaphragmatic activity could restore diaphragmatic thickness. Secondarily, we aim to correlate the change in diaphragm thickness during both CMV and AMV to clinical factors and outcomes. As the third objective, we aim to describe the boundaries of “diaphragm-protective assisted ventilation” according to the clinical outcomes of our patients and to correlate diaphragm activity to thickness.

## Methods

This is a prospective observational study performed in the general ICUs of San Gerardo Hospital in Monza and Desio Hospital, Cernusco S/N Hospital, Niguarda Hospital, and Ospedale Maggiore Policlinico in Milan.

The study protocol was approved by the local Ethics Committees of the involved hospitals, and informed consent was obtained from patients or their substitute decision-makers prior to enrolment.

The participating centres managed patients in the acute phase of the critical illness with CMV (with or without muscle paralysis but avoiding spontaneous breathing effort), switching to AMV after improvement of the clinical condition (i.e. resolution of acute phase, FiO_2_ < 50%, reduction in the doses of vasopressors), typically by PSV. PS level was progressively reduced until a phase of continuous positive airway pressure (cPAP) ventilation. In this context, it is possible to clearly separate the fully controlled or assisted ventilation mode and to identify the onset of inspiratory activity.

### Inclusion and exclusion criteria

We enrolled intubated or tracheotomized patients undergoing controlled mechanical ventilation for at least 48 cumulative hours (with intervals of assisted ventilation of less than 24 h).

Exclusion criteria were age lower than 18 years old, body mass index (BMI) greater than 35, pregnancy, neuromuscular diseases, phrenic nerve lesions, abdominal vacuum-assisted closure (VAC) therapy, and poor acoustic window.

Patients were subsequently excluded from the final analysis if they were not switched to assisted mechanical ventilation or if the total period of mechanical ventilation was shorter than 8 days (less than four US measurements available).

### Study protocol

At the time of enrolment, age, sex, body mass index (BMI), comorbidities, reason for admission, therapy with corticosteroids or neuromuscular blocking agents (NMBA), ventilator parameters (tidal volume, respiratory rate, PEEP, pressure support level), haemodynamic and gas exchange parameters, SOFA score, and nutritional status indicators (such as prealbumin levels [16, 17]) were collected, and US was performed. Glasgow Coma Scale was not taken into account during the computation of SOFA. Some derived data were computed to describe patients’ respiratory mechanics. Driving pressure was computed as the difference between plateau pressure (measured during an inspiratory hold, both in CMV and AMV, as previously described by our group [10, 18]) and PEEP. Respiratory system compliance was calculated as tidal volume divided by driving pressure. Pressure Muscle Index (PMI) is the difference between plateau pressure and peak pressure (the latter being the sum of pressure support and PEEP) during PSV and was previously shown to correlate with the effort exerted by patients’ muscle during inspiration [19]. Lastly, Rapid Shallow Breathing Index (RSBI) is the product between tidal volume and respiratory rate, and it is used to evaluate weaning readiness [20]. p0.1 [21], the drop in pressure in the first 100 ms of an inspiratory effort against occluded airways and an index of respiratory drive, automatically measured by the mechanical ventilators, was collected together with the other ventilator data during AMV.

The linear US transducer was placed in the IX or X intercostal space near the midaxillary line and angled perpendicular to the chest, as validated by Goligher et al. [22]. The diaphragm was identified as a three-layered structure just superficial to the liver, consisting of a relatively non-echogenic muscular layer bounded by the echogenic membranes of the diaphragmatic pleura and peritoneum (Additional file 1: Figure S1A). The zone of apposition of the diaphragm between the liver and chest wall was identified. The first ultrasound image of diaphragm was collected at the end of expiration in two-dimensional (2D) mode (Additional file 1: Figure S1A ). The second scan was collected in motion mode (M-mode) with at least two breaths visualized (Additional file 1: Figure S1B). A mark with a skin marker was made at the point of the first measurement to improve the reproducibility of the measurements, as previously demonstrated [22].

Every 48 h, ultrasound images and clinical data collection were repeated until discharge from the intensive care unit or death.

US measurements were stored during all procedures for subsequent offline analysis of diaphragm thickness and diaphragm thickening fraction.

**Fig. 1:**
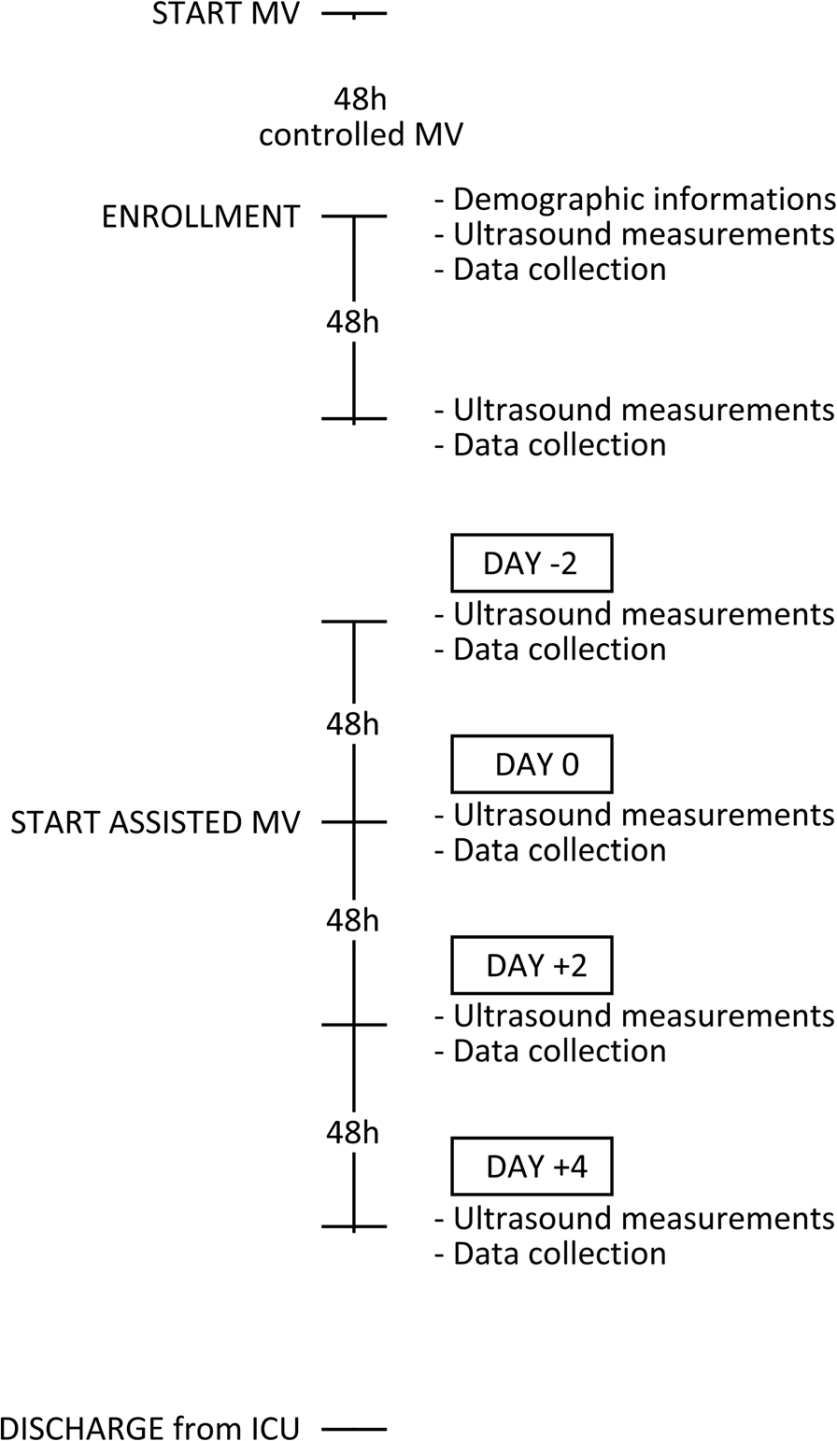
Timeline of the study protocol. The day in which the patient was switched from controlled to assisted mechanical ventilation was marked as day 0. The study days before day 0 were numbered with negative numbers and correspond to the controlled ventilation days; the days after day 0 were numerated with positive numbers and correspond to the assisted mechanical ventilation period. MV, mechanical ventilation; ICU, intensive care unit

In the following analysis, we refer to the data collection day closest to the first day of assisted MV as “day 0”. Therefore, we assign a negative value to the data collection days before “day 0” (corresponding to CMV days) and a positive value to the data collection days after “day 0” (corresponding to AMV days). The study protocol is summarized in Fig. 1.

### Offline analysis

Offline analyses of US images were performed with the support of a dedicated software (ImageJ®, US National Institutes of Health, Bethesda, MD, USA).

Diaphragm thickness (Tdi) was measured in 2D mode images (performed at end-expiration) as the distance between the diaphragmatic pleura and the peritoneum (Additional file 1: Figure S1A). Tdi was expressed as a percentage of Tdi in day 0.

In M-mode images, diaphragm thickness was measured at end expiration (Tdi,ee) and at inspiratory peak (Tdi,pi) (Additional file 1: Figure S1B ). Diaphragm thickening during inspiration (DTdi) was calculated as the difference between Tdi,pi and Tdi,ee. Diaphragm thickening fraction (TFdi) was defined as the percentage change in diaphragm thickness during inspiration and computed as the quotient of DTdi and Tdi,ee.

### Aims of the study

The first aim of this study was to verify the hypothesis that the mode of ventilation correlates with changes in diaphragm thickness. More specifically, we hypothesize that, while CMV leads to diaphragm atrophy, AMV can lead to partial or total restoration of diaphragm thickness.

The second aim was to correlate the change in diaphragm thickness (both as thinning during CMV and as recovery of thickness during AMV) to clinical outcomes, namely, ICU and hospital survival, ICU and hospital length of stay, and duration of mechanical ventilation. The third aim of the study was to correlate the changes in diaphragm thickness with clinical factors such as comorbidities, admission diagnosis, severity of disease, ventilation settings and respiratory mechanics, and prealbumin as a nutritional index. Lastly, we aimed to correlate the change in diaphragm thickness to diaphragm function, expressed as diaphragm thickening fraction and to respiratory drive and effort.

### Statistical analysis

Statistical analysis was performed with SPSS Statistics® v. 25.0 (IBM Corporation, Armonk, NY, USA).

Descriptive statistics were expressed as means and standard deviation or standard error for variables with a normal distribution, otherwise as medians [interquartile range (IQR)]. Normality was assessed by the Shapiro-Wilk test.

We used linear mixed models to evaluate the changes in diaphragm thickness and thickening fraction over time.

The study population was divided into two groups based on diaphragm change in thickness during CMV period. A change of at least 10% (as compared to Tdi in day 0) in at least one US measurement between study enrolment and day 0 (switch to AMV) was used to define two groups: “unchanged during CMV” and “thinning during CMV”. Similarly, the study population was then divided into two groups based on a change of at least 10% (as compared to day 0) in at least one of the US measurements taken between day 0 (start of AMV) and the last US measurement available (death, discharge from ICU or ventilation switched back to CMV). The two groups were defined as “unchanged during AMV” and “recovery of thickness during AMV”.

The 10% cut-off selection was based on the measurement resolution of the ultrasound technique [23] and on the previously published study in which this cut-off was used [2].

Clinical characteristics and changes in diaphragm function were compared among patients in (1) “unchanged during CMV” versus “thinning during CMV” and (2) “unchanged during AMV” and “recovery of thickness during AMV”, using independent samples *t* test, Mann-Whitney test, or chi-square test as appropriate. Total days of neuromuscular blocker infusion were normalized for total days of controlled ventilation before performing a statistical analysis. Linear correlation analysis was assessed using Pearson’s or Spearman’s model, based on the normal distribution of data.

To compare the percentages of survivors between the groups, we also used Cohen’s *h* measure of effect size between two independent proportions [24].

The estimation of the sample size was based on the following considerations:

- The aim of the study is to evaluate the change in diaphragmatic thickness and inspiratory diaphragm thickening in patients undergoing assisted ventilation after at least 48 h of controlled ventilation.
- The average reduction in diaphragm thickness in ventilated patients during the first week is 20 ± 10% [2, 25, 26].
- We expect a variation of this diaphragmatic reduction of 20% by switching to assisted ventilation.
- In order to prove this result with an alpha risk of 0.05 and a power of 80%, 50 patients are needed. Because the diaphragm cannot be visualized by US in about 10% of patients and another 10% of patients will not be weaned from controlled ventilation, we planned to enrol 60 patients in total.

## Results

### Population selection

**Fig. 2:**
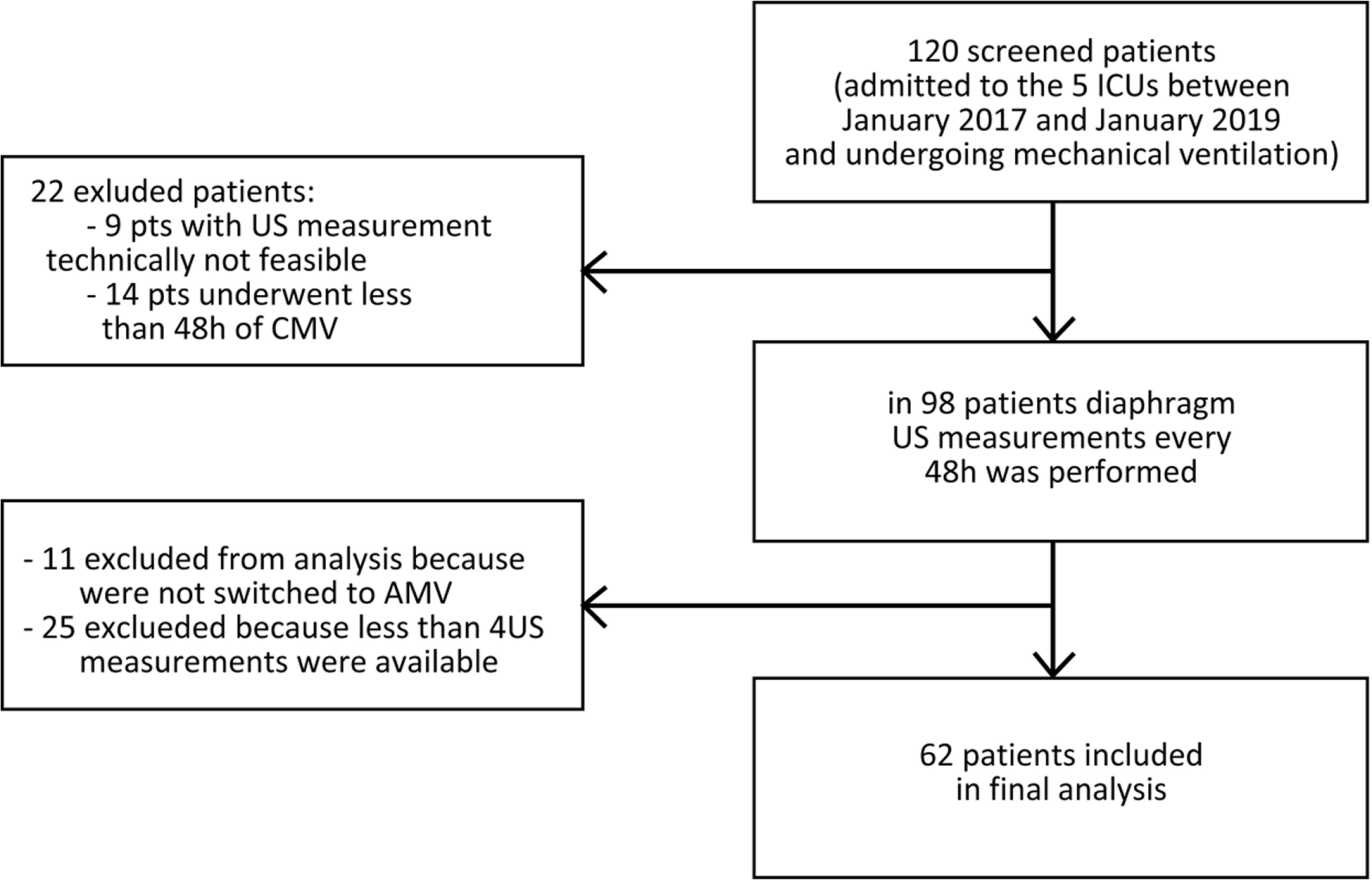
Population selection. The population selection process which led to the final study population of 62 patients. ICU, intensive care unit; US, ultrasound; CMV, controlled mechanical ventilation; AMV, assisted mechanical ventilation

One hundred twenty ICU patients were screened between January 2017 and January 2019. After the application of exclusion criteria, 98 patients were included in the study. Thirty-six of them dropped out because they were never switched to assisted ventilation (*n* = 11 patients died during CMV period) or were ventilated for a short period of time (*n* = 25 patients had less than 4 US measurements available). Sixty-two of them were included in the final analysis after undergoing a period of controlled ventilation followed by weaning through assisted ventilation (Fig. 2). The characteristics of the population at baseline are shown in Additional file 1: Table S1.

A mean of 9.6 ± 5.5 US measurements was taken for every patient until ICU discharge. The analysis was performed on the US measurements and clinical data collected between the day of enrolment and day + 8 of mechanical ventilation, when the highest number of data collected was available.

### Thickening fraction over time

**Fig. 3:**
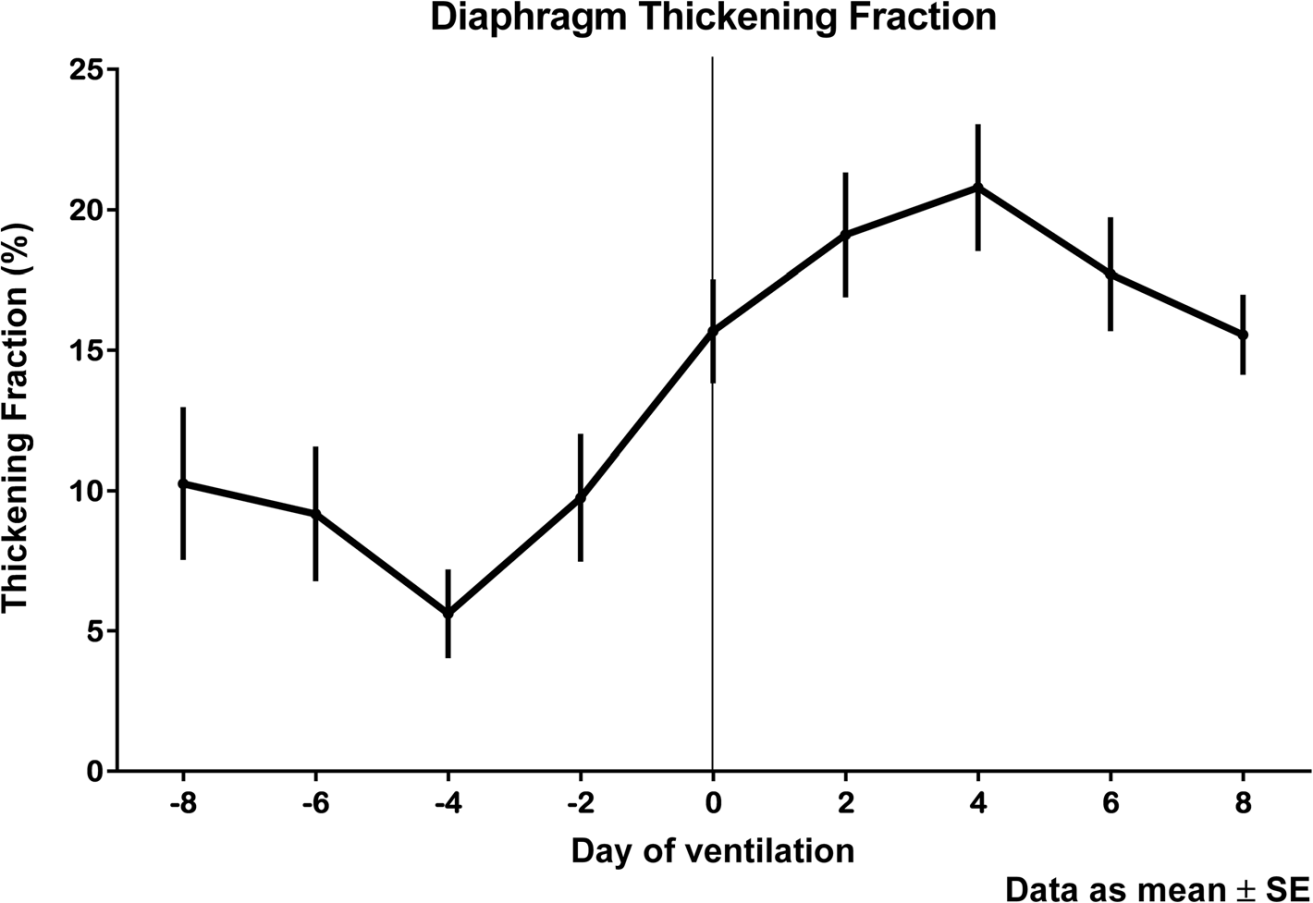
Diaphragm thickening fraction over time. Diaphragm thickening fraction was lower during the days before day 0 (corresponding to the time spent by the patient ventilated in controlled modes) than during the days after day 0 (corresponding to the period spent with assisted ventilation modes)

TFdi, which reflects diaphragmatic contraction, as expected, significantly increased during AMV versus CMV period: in fact, mean TFdi during CMV was 7.2 ± 6.8%, while the mean TFdi during AMV was 17 ± 9.5%, *p* < 0.0001. Figure 3 shows the change of mean TFdi over time.

### Average change in diaphragmatic thickness according to ventilation mode

**Fig. 4:**
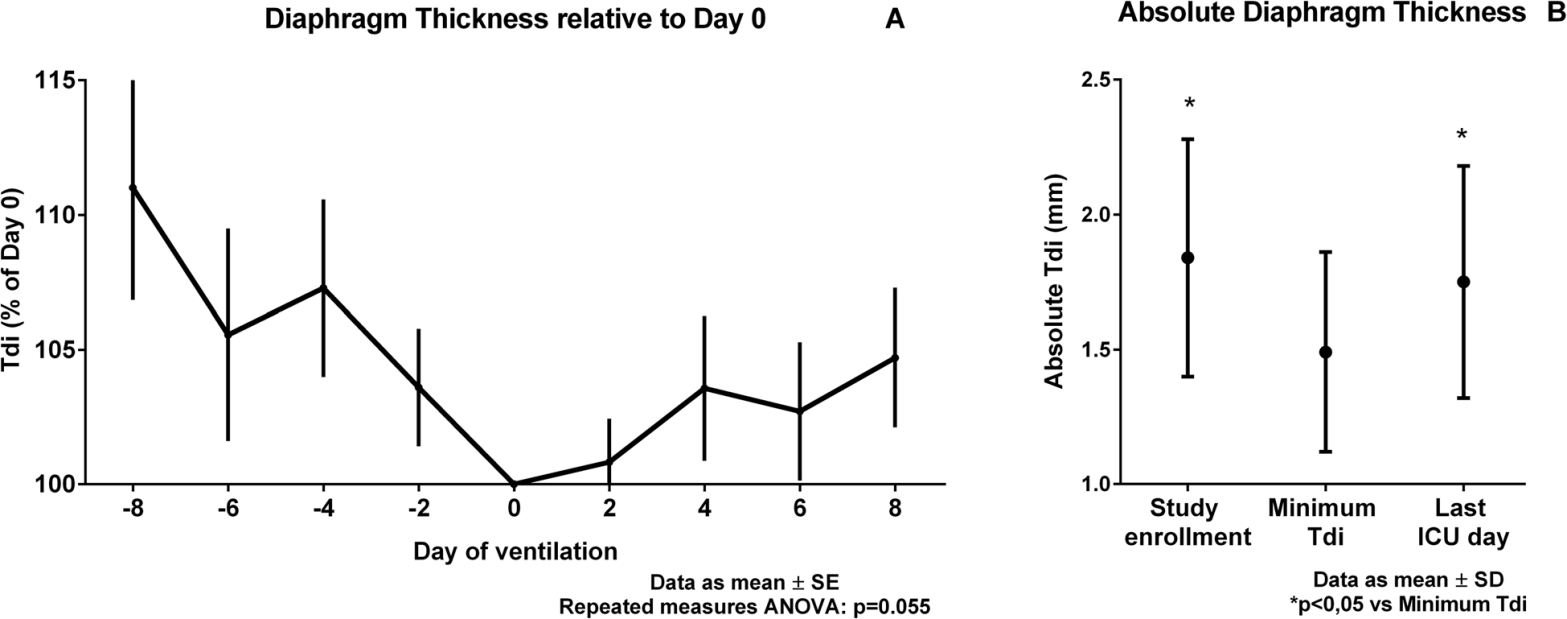
Diaphragm thickness over time. **a** The change in diaphragm thickness during the course of mechanical ventilation. Thickness is expressed as the percentage of the thickness in day 0 for every patient. Day 0 marks the switch from controlled to assisted mechanical ventilation mode. While during controlled ventilation (days before day 0), diaphragm thickness tends to decrease, during assisted ventilation partial restoration of the muscle thickness occurs. **b** The mean change in absolute diaphragm thickness from the beginning to the end of the study period. Tdi, diaphragm thickness; CMV, controlled mechanical ventilation; ICU, intensive care unit; SE, standard error; SD standard deviation

CMV was associated with a decrease in diaphragm thickness while during the period of AMV, it partially recovered (Fig. 4a). When taking into consideration the first 2 days of CMV and the last 2 days of ICU stay in each patient, the decrease and subsequent increase in diaphragm thickness were both significantly different when compared to diaphragm thickness in day 0 (Additional file 1: Figure S2). The same results were obtained looking at absolute Tdi values at the beginning and at the end of the study period: the mean absolute thickness at the study enrolment was 1.84 ± 0.44 mm, while the last value of thickness recorded was 1.75 ± 0.43 mm. The minimal thickness value reached was 1.49 ± 0.37 mm, significantly different from both the values recorded at the beginning and at the end of the study period (*p* < 0.001) (Fig. 4b).

The mean Tdi reduction from the study enrolment to the day of switch to AMV was 11.2 ± 21.6%.

### Change in diaphragmatic thickness and patients’ outcome

**Fig. 5:**
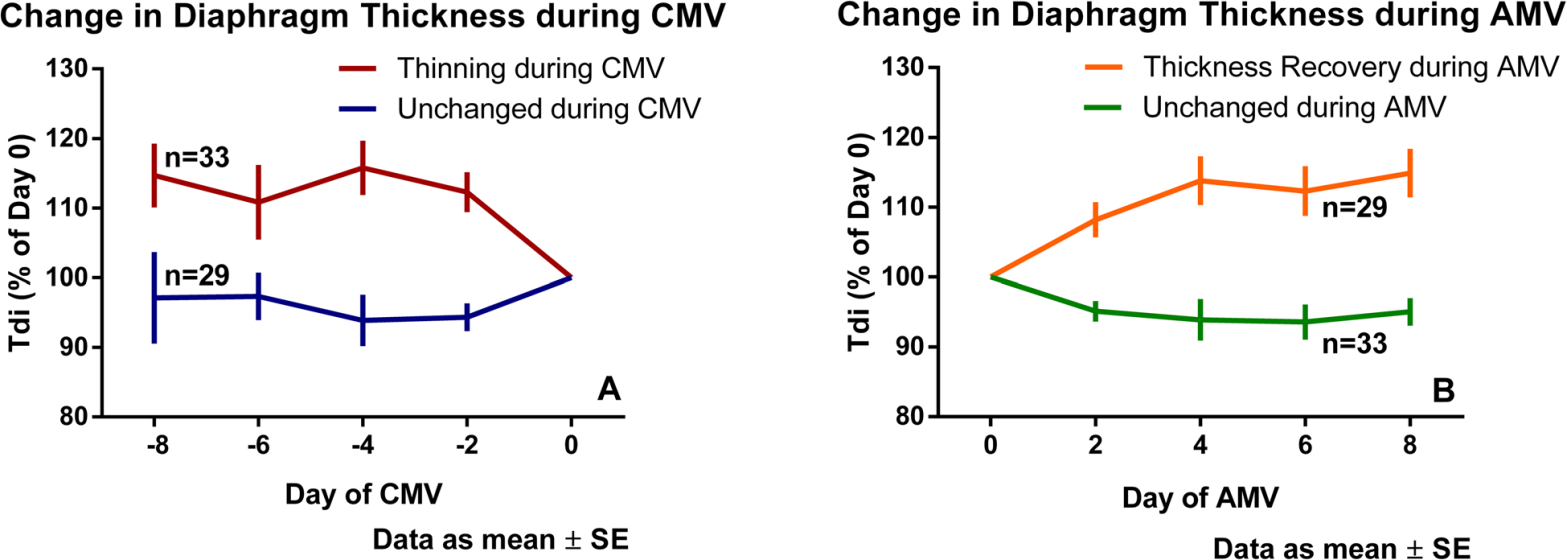
Classification of patients based on the changes in diaphragmatic thickness during controlled and assisted mechanical ventilation. **a** Patients were classified into two groups based on the thinning of the diaphragm during controlled ventilation. Patients whose diaphragm thickness decreased by more than 10% are represented by the red line; patients whose diaphragm was unchanged are represented by the blue line. **b** Patients were classified into two groups based on the thickening of the diaphragm during assisted ventilation. Patients whose diaphragm thickness increased by more than 10% are represented by the orange line; patients whose diaphragm was unchanged are represented by the green line. CMV, controlled mechanical ventilation; AMV assisted mechanical ventilation: Tdi, diaphragm thickness; SE, standard error

Patients were classified into two groups based on the change in diaphragm thickness during CMV (Fig. 5a). A decrease in diaphragm thickness by 10% or more was associated with significantly more days spent under CMV, absolute and as a percentage of the total ventilation period. Diaphragm thinning was not associated with different ICU or hospital outcome nor different length of stay, as also shown by the small effect size for survival differences (close to 0.2) with a wide confidence interval (Table 1).

**Table 1:** Change in diaphragm thickness and outcomes during controlled and assisted mechanical ventilation

	**Unchanged during CMV (*n*** **= 29)**	**Thinning during CMV (*n*** **= 33)**	***p***	**Cohen’s** ***h*** **(95% CI)**
ICU survival, *n* (%)	25/29 (86.2%)	31/33 (93.9%)	0.304	0.264 [− 0.235, 0.762]
Hospital survival, *n* (%)	25/29 (86.2%)	31/33 (93.9%)	0.304	0.264 [− 0.235, 0.762]
CMV (days)	5.0 [4.0, 8.5]	10.0 [5.0, 15.0]	0.004	
AMV (days)	10.0 [6.0, 17.0]	8.0 [4.0, 13.0]	0.390	
CMV days on total MV (%)	34.8 [27.9, 50.0]	55.6 [39.4, 71.8]	0.001	
ICU length of stay (days)	21.0 [14.5, 27.0]	23.0 [15.0, 36.0]	0.311	
	**Unchanged during AMV (*n*** **= 33)**	**Recovery of thickness during AMV (*n*** **= 28)**	***p***	
ICU survival, *n* (%)	31/33 (93.9%)	25/28 (89.3%)	0.509	− 0.169 [− 0.673, 0.334]
Hospital survival, *n* (%)	31/33 (93.9%)	25/28 (89.3%)	0.509	− 0.169 [− 0.673, 0.334]
CMV (days)	8.0 [4.5, 13.0]	8.0 [5.0, 13.0]	0.822	
AMV (days)	9.0 [5.0, 16.0]	10.0 [4.3, 14.8]	0.913	
AMV days on total MV (%)	55.0 [42.0, 68.0]	50.0 [31.0, 71.0]	0.519	
ICU length of stay (days)	22.0 [14.0, 34.0]	23.0 [16.0, 29.0]	0.812	

The top part of the table shows the association between diaphragm changes and outcomes during CMV. The bottom part of the table shows the same association during AMV

*ICU* intensive care unit, *CMV* controlled mechanical ventilation, *AMV* assisted mechanical ventilation

Subsequently, patients in the AMV period were classified based on a regain of diaphragm thickness of more or less than 10% (Fig. 5b). The two groups of patients were not different for any of the outcomes tested (survival and duration of ventilation and length of ICU staying in survivors, Table 1).

### Clinical factors associated with changes in diaphragm thickness

All the clinical factors associated with diaphragm thinning during CMV are shown in Table 2. These patients underwent mechanical ventilation with higher PEEP and higher plateau pressure. They had also a trend towards more days spent under NMBA. Lastly, lower mean prealbumin levels in the plasma during ICU admission were associated with decreased diaphragm thickness during CMV.

**Table 2:** Clinical factors associated with the change in diaphragm thickness during controlled mechanical ventilation

	Unchanged during CMV (*n* = 29)	Thinning during CMV (*n* = 33)	*p*
Age (years)	56.0 ± 16.2	55.7 ± 14.8	0.945
Body weight (kg)	78.9 ± 11.8	72 ± 12	0.033
BMI (kg/cm^2^)	26.2 ± 3.3	25 ± 3.8	0.197
Chronic obstructive pulmonary disease, *n* (%)	2/29 (6.9%)	4/33 (12.1%)	0.488
Diabetes, *n* (%)	2/29 (6.9%)	7/33 (21.2%)	0.110
ARDS, *n* (%)	10/29 (34.5%)	15/33 (45.5%)	0.380
Septic shock, *n* (%)	6/29 (20.7%)	5/33 (15.2%)	0.569
NMBA (days)	0 [0, 4]	3 [0, 6.5]	0.127
NMBA days on CMV days (%)	28.7 [0, 56.6]	35.7 [0, 83.8]	0.456
Mean SOFA during ICU admission	4.8 [3.4, 6.5]	4.4 [3.3, 5.5]	0.399
TV/kg of ideal body weight	6.6 ± 1.4	6.7 ± 1.5	0.968
Respiratory rate during CMV (bpm)	14 [11.5, 19.3]	15 [11.5, 20.1]	0.601
Peak pressure during CMV (cmH_2_O)	26.4 ± 5.7	27.5 ± 5.7	0.518
PEEP during CMV (cmH_2_O)	10.4 ± 4	12.6 ± 4	0.034
Plateau pressure during CMV (cmH_2_O)	21.9 ± 3.9	23.9 ± 3.5	0.063
Driving pressure during CMV (cmH_2_O)	10.6 ± 2.9	10.6 ± 1.9	0.948
Compliance of respiratory system during CMV (cmH_2_O)	44.5 [32.5, 50]	44.1 [32.4, 48.8]	0.887
P/F during CMV	212 [142, 261]	189 [156, 236]	0.527
pH during CMV	7.42 ± 0.04	7.42 ± 0.02	0.476
Total water balance during CMV	1699 [− 1109, 4240]	1148 [− 1568, 3151]	0.305
Prealbumin (mg/dl)	23.4 ± 7.6	18.5 ± 6.6	0.050

*BMI* body mass index, *ARDS* acute respiratory distress syndrome, *NMBA* neuromuscular blocking agents, *CMV* controlled mechanical ventilation, *SOFA* Sequential Organ Failure Assessment, *ICU* intensive care unit, *AMV* assisted mechanical ventilation, *TV* tidal volume, *PEEP* positive end-expiratory pressure

No differences in previous diseases nor in the main diagnosis for hospital admission were associated with regain of diaphragm thickness during AMV. This was instead associated with lower respiratory rate and RSBI and with higher PMI and a trend towards higher p0.1 (Table 3).

**Table 3:** Clinical factors associated with the change in diaphragm thickness during assisted mechanical ventilation

	Unchanged during AMV (*n* = 33)	Recovery of thickness during AMV (*n* = 28)	*p*
Age (years)	53.2 ± 15.8	58.4 ± 14.4	0.187
Body weight (kg)	76.4 ± 12.6	74.3 ± 13	0.524
BMI (kg/cm^2^)	25.8 ± 3.3	25.4 ± 3.9	0.681
Chronic obstructive pulmonary disease, *n* (%)	2/33 (6.1%)	3/28 (10.7%)	0.509
Diabetes, *n* (%)	2/33 (6.1%)	5/28 (17.9%)	0.150
ARDS, *n* (%)	14/33 (42.4%)	11/28 (39.3%)	0.804
Septic shock, *n* (%)	3/33 (9.1%)	7/28 (25.0%)	0.094
NMBA (days)	2 [0, 5.5]	2 [0, 5]	0.774
NMBA days on CMV days (%)	28.6 [0, 75.7]	30.4 [0, 85.6]	0.803
Median SOFA during ICU admission	4.6 [3.2, 5.9]	4.3 [3.4, 5.3]	0.606
TV/kg of ideal body weight	6.9 [6.1, 7.8]	7.4 [6.4, 8.5]	0.152
Respiratory rate during AMV (bpm)	19.2 ± 4	16.7 ± 3.2	0.019
Rapid Shallow Breathing Index (RR/TV, breaths/min/l)	44 ± 13	37 ± 11	0.029
Peak pressure during AMV (cmH_2_O)	17.9 ± 4.1	17.1 ± 4.1	0.441
Pressure support level (cmH_2_O)	8.4 ± 2.4	7.6 ± 2.4	0.163
PEEP during AMV (cmH_2_O)	8.9 ± 2.5	8.9 ± 2.4	0.984
Plateau pressure during AMV (cmH_2_O)	20.1 ± 3.1	18.9 ± 3.9	0.225
Driving pressure during AMV (cmH_2_O)	10.2 ± 2.6	9.5 ± 3	0.459
Compliance of respiratory system during AMV (cmH_2_O)	52.5 ± 17.4	54.8 ± 14.4	0.618
Negative inspiratory force (cmH_2_O)	− 15.5 [− 21.8, − 8.4]	− 16.8 [− 26.5, − 11.5]	0.451
Pressure Muscle Index (cmH_2_O)	0.4 [0, 1.9]	2 [0.5, 3]	0.024
P0.1 (cmH_2_O)	1.2 ± 0.8	1.8 ± 1.5	0.064
Thickening fraction (%)	15 [8, 23.8]	19.1 [9.2, 25.7]	0.397
P/F during AMV	242.3 ± 51	270 ± 82.1	0.159
pH during AMV	7.46 [7.44, 7.48]	7.45 [7.43, 7.48]	0.397
Total water balance during AMV	− 5759 [− 9632, − 1348]	− 4884 [− 10742, − 2460]	0.869
Prealbumin (mg/dl)	18.8 [13.5, 26]	22.5 [18.9, 24.4]	0.438

*BMI* body mass index, *ARDS* acute respiratory distress syndrome, *NMBA* neuromuscular blocking agents, *CMV* controlled mechanical ventilation, *SOFA* Sequential Organ Failure Assessment, *TV* tidal volume, *AMV* assisted mechanical ventilation, *PEEP* positive end-expiratory pressure

### Factors associated with diaphragm activity

In 11 out of 62 patients (18%), the measurement of TFdi on M-mode US images was considered not feasible. Therefore, the following results refer to the remaining 51 patients. The median TFdi of the patient population during the first 8 US measurements in AMV was 16.3% [IQR 8.9, 24.6]. The patients with a mean TFdi higher than 16.3% had a higher incidence of ARDS as admission diagnosis (54% had ARDS at admission versus 16% in the group whose mean TFdi was < 16.3%, *p* < 0.005). The median of the maximum TFdi recorded for each patient during the first 8 US measurements during AMV was 25% [IQR 14, 35]. We then classified the patients in tertiles of mean TFdi during the same period of AMV (corresponding to the first 8 US measurements): despite the differences between the groups were not significant, we found that with the increase in mean TFdi, the AMV days (so the weaning duration) increased, together with ICU length of stay (Additional file 1: Figure S3).

### Relation between diaphragmatic thickening fraction, diaphragmatic thickness and indexes of respiratory drive and effort

We did not find a significant relationship between the level of inspiratory effort expressed as TFdi and the mean and maximum thickness reached by the diaphragm during the AMV period. The mean and maximum diaphragm thickness were also not related to other indices of inspiratory effort, such as mean PMI, p0.1, and negative inspiratory force (NIF) (Additional file 1: Figure S4 A and B).

## Discussion

While diaphragm atrophy is widely described in the recent literature, the reversal of this process is still poorly studied and it is not clear which factors can favour it. Moreover, the increase of diaphragm thickness described during some phases of mechanical ventilation might not represent a recovery of the muscle mass but the occurrence of a pathological process. The purpose of this study was to partially address these open questions by looking at the effect of assisted mechanical ventilation on diaphragm thickness and activity and their relationship to patients’ outcome.

The main results can be summarized as follows: diaphragm thickness is influenced by the mode of ventilation; in fact, while during CMV, Tdi tends either to decrease or not change, spontaneous assisted breathing with pressure support can lead to partial restoration of Tdi. A decrease in Tdi during CMV is associated with a longer period of diaphragm inactivity (duration of CMV), while a regain in Tdi during AMV in our population was not associated with common clinical outcomes. Considering diaphragm activity, the patients with lower TFdi trended towards less AMV days. Lastly, we found a correlation between the increase in Tdi during AMV and lower respiratory rate and lower Rapid Shallow Breathing Index as well as higher Pressure Muscle Index, whereas we found no association between diaphragm thickness and activity, as measured by TFdi.

Our data confirm that diaphragm atrophy occurs in ICU patients, and this can be captured by US [27]. The mean thickness value recorded at the study inclusion was similar to that recorded in another study [25]. A decrease in Tdi was shown to be associated with longer periods of mechanical ventilation, and it occurs early after intubation [2, 25]. The higher levels of PEEP in patients with diaphragm thinning during CMV could be in line with the recent evidences regarding myofibre longitudinal atrophy due to PEEP [6]. To our knowledge, no studies until now described the relation between the prealbumin levels, as an index of nutritional status [17], and diaphragm thickness. If it can be argued that both lower prealbumin and diaphragm thinning are simply a marker of more severe baseline disease, this is not confirmed by differences in baseline SOFA or incidence of shock in the two groups (thinning or unchanged diaphragm during CMV). Therefore, these observational data can give a suggestion regarding a possible preventive approach to avoid diaphragm thinning by means of monitoring and optimization of nutritional status.

While the negative effects of prolonged diaphragm inactivity are widely recognized, clinical data regarding the consequences of partial diaphragm activity, which happens during assisted modes of ventilation, are less clear and somehow conflicting.

Animal studies clearly showed that different modes of assisted ventilation induce a milder loss of diaphragm function [14, 28] while also causing less proteolysis [13].

On the clinical side, instead, the first important factor to be underlined is that, because of the ventilation practice of different centres, the period of controlled and assisted ventilation are not clearly dissectable, and therefore, it is difficult to correlate the trend of diaphragm thickness with a specific mode of ventilation [25]. Two Italian studies focused specifically on the consequences of the mode of ventilation on the diaphragm. Umbrello et al. [29] showed that the level of diaphragm activity was related to the level of support provided by the ventilator and to other indices of respiratory effort (p0.1, PMI), but given the short duration of patient observation in this study, it lacks a trend of Tdi measurements overtime. Zambon et al. [26] instead correlated Tdi with the ventilation setting and, in keeping with our results, demonstrated that the daily atrophy rate of the diaphragm is directly related to the mode of ventilation; partial preservation of diaphragm activity during PSV or spontaneous breathing with cPAP significantly reduced the atrophy rate. In 2015, Goligher et al. [2] showed a direct relation between diaphragm thickness and activity, expressed as TFdi. In a supplementary analysis of this study, patients were classified based on the mode of ventilation (controlled versus partially assisted): in both cases, the diaphragm thickness decreased at the beginning of ventilation time, then it tended to increase only in the “partially assisted mode” patients, despite not significantly. The relation between diaphragm Tdi, diaphragm activity, and duration of mechanical ventilation shown in Goligher’s study [2] was not confirmed by our results; this could be due by the fact that, on average, the Tdi in our patients tended to decrease and then to return to baseline. An intriguing very recent study [15] shows that diaphragm partial activity during mechanical ventilation preserves muscle fibres from histological damage, contrarily to muscle complete inactivity. We have no histological data on patients’ diaphragms, and further studies, maybe involving muscular structure evaluation, are needed to judge if the muscle actually benefits from the assisted activity, even if in some cases, this might not translate in improved outcome due to the co-existence of too many confounding factors in an ICU patient.

Regarding diaphragm activity, some studies report that the level of TFdi can be used as a predictor of successful weaning [9, 30, 31]. This evidence was not confirmed in other studies. One of them found TFdi to be less sensitive and specific of traditional measure of breathing effort and distress [32]. Another recent study [33] looked specifically at extubation failure in those patients who successfully passed a spontaneous breathing trial (SBT). In this population, neither TFdi nor diaphragm excursion was predictors of successful extubation, and the only factor associated with successful extubation was effective cough. Sixty-three percent of the patients who passed the SBT and were successfully extubated had a TFdi of the right diaphragm lower than 30%.

Similarly, we found low values of mean and maximum TFdi during the period of AMV in our patients. First, we focused on tidal TFdi, while in other studies, TFdi is calculated between maximum inspiration and expiration [2, 34, 35]. Second, in a larger population of ventilated patients, the majority of the subjects (128 out of 146) had a TFdi lower than 25% during the first 3 days of ventilation [1]. Third, the low TFdi that we recorded somehow reflects the weaning approach used in the centres involved in the study. All the centres are geographically close and traditionally use PSV as the main mode to wean patients from a mechanical ventilator. Spontaneous breathing trials are usually performed in a cPAP mode with a level of PEEP < 10 cmH_2_O and only after a gradual decrease of the level of pressure support, which could happen along many days. The amount of inspiratory effort is usually measured more than once per day, by means of p0.1 and PMI. PEEP is typically set by the clinician based both on respiratory system compliance and oxygenation response [36], taking also into account that the level of PEEP itself can modify the respiratory effort of the patient [37]. Therefore, it is very uncommon that patients’ endurance is tested causing strenuous levels of inspiratory effort, as also witnessed by the low median Rapid Shallow Breathing Index in all the patients. TFdi is normally not monitored by the clinician. These results could help delineate the parameters and safe thresholds for “diaphragm-protective mechanical ventilation”, and once again, they underline the importance of monitoring the level of inspiratory effort in spontaneously breathing patients and the need to avoid strenuous efforts. In fact, a trend towards longer mechanical ventilation and longer ICU stay was observed in those patients whose mean TFdi was higher in the first days of the weaning process. This could suggest that keeping the diaphragm at rest, with low levels of TFdi, promotes rehabilitation of the muscle. Or that, in patients who are healthier, and therefore ventilated for a shorter time, a low TFdi is enough to promote successful weaning from the mechanical ventilator.

RSBI is a commonly used index to evaluate extubation readiness during weaning [20], whereas PMI (namely the pressure recorded on the ventilator during an inspiratory hold in PSV) was shown to tightly correlate with inspiratory pressure generated by the patient’s muscles during inspiration [19]. We found a correlation between an increase in diaphragm Tdi during AMV and lower RSBI; the presented data do not allow to infer any causal relationship between these two phenomena, but for the first time, we show that a respiratory pattern as close as possible to normal breath happens at the same time as a partial restoration in diaphragmatic thickness. On the other side, the correlation between recovery of thickness and PMI could support the hypothesis that muscle thickness restoration brings to a higher force generation, leading to a trend towards higher tidal volumes despite lower mean pressure support levels (Table 3). All these data point towards the fact that diaphragm thickness recovery during AMV is a different process when compared to diaphragm absolute increase in thickness, as described by Goligher et al. [2], the latter correlated with diaphragm dysfunction. In our patient, restoration of diaphragm thickness through a smooth training during AMV was associated with a more efficient diaphragm function. This observation could be hypothesis-generating for a subsequent study.

The main strength of this study lies in the ability of clearly separating the phases in which patient’s inspiratory effort is present or absent, enabling to investigate the specific consequences of the mode of ventilation on diaphragm thickness and activity. We acknowledge that the consequences of ventilation on myotrauma need to be further analysed [38], even if a “lung-protective ventilation” during assisted modes, which mainly consists in the strict monitoring of respiratory mechanics and effort is the main key to improve outcome [10] bringing also to a smooth training of the diaphragm while weaning the lungs.

### Study limitations

The first one is related to the US method per se; in fact, US diaphragm images taken in ICU patients are influenced by dozens of factors, even if all the attention was paid to guarantee reproducible measures [22]. This lead to discarding 18% of the TFdi measurement that were judged to be unreliable. Secondarily, the multicentre nature of this study leads to a higher population heterogeneity. Third, the population suffers from a selection bias, which was however wanted by the investigators, in order to focus on those patients who are considered able to be weaned (so switched from CMV to AMV). This explains the high survival rate observed in this ICU population. Forth, we acknowledge that we performed a high number of statistical analysis, which could lead to associations difficult to interpret, even if we repeated most of the analysis already performed in previous studies [2].

## Conclusion

The mode of ventilation affects diaphragm thickness in ICU patients. While diaphragm atrophy is associated with a longer duration of controlled mechanical ventilation, assisted mode leads to partial restoration of diaphragm thickness, which seems to correlate to a better breathing pattern. These findings on one side underline the need for monitoring respiratory mechanics during spontaneous breathing and avoiding excessive efforts, on the other side provide some data to define the thresholds for “diaphragm-protective mechanical ventilation”.

## Supplementary information

**Additional file 1.** Supplementary figures and tables.

## Data Availability

The datasets used and/or analysed during the current study are available from the corresponding author on reasonable request.

## Abbreviations

AMV: Assisted mechanical ventilation
ARDS: Acute respiratory distress syndrome
BMI: Body mass index
CMV: Controlled mechanical ventilation
cPAP: Continuous positive airway pressure
DTdi: Diaphragm thickening during inspiration
ICU: Intensive care unit
IQR: Interquartile range
NMBA: Neuromuscular blocking agents
NIF: Negative inspiratory force
PEEP: Positive end-expiratory pressure
PMI: Pressure Muscle Index
PSV: Pressure support ventilation
RSBI: Rapid Shallow Breathing Index
SD: Standard deviation
SE: Standard error
SOFA: Sequential Organ Failure Assessment
Tdi: Diaphragm thickness
Tdi,ee: Diaphragm thickness at end expiration
Tdi,pi: Diaphragm thickness at peak inspiration
TFdi: Diaphragm thickening fraction
US: Ultrasound
VAC: Vacuum-assisted closure
