# Supplementary material for: Assisted mechanical ventilation promotes recovery of diaphragmatic thickness in critically ill patients: a prospective observational study
Source: Crit Care. 2020 Mar 12;24:85. doi: 10.1186/s13054-020-2761-6 (PMC7068963; doi:10.1186/s13054-020-2761-6)
Supplement: Supplementary file 1 — Additional file 1. Supplementary figures and tables. [file 13054_2020_2761_MOESM1_ESM.docx]

**ADDITIONAL FIGURES AND TABLE**

**Figure S1. Measurement of diaphragmatic thickness and thickening fraction**

Panel A shows a representative image of diaphragm thickness measurement in Bmode. Panel B shows a representative image of diaphragm thickening fraction measurement in Mmode. Tdi,pi diaphragm thickness at peak inspiration. Tdi,ee diaphragm thickness at end expiration.

**Table S1. Demographic and clinical characteristics of study population at baseline**

|  |  |
| --- | --- |
| Age (years, mean ± SD) | 56 ± 15 |
| Sex, number of males (%) | 44 (71%) |
| Body weight (kg, mean ± SD) | 75 ± 13 |
| Body Mass Index (kg/cm^2^, mean ± SD) | 25.5 ± 3.6 |
| SOFA at admission (GCS NOT INCLUDED), mean ± SD | 7.6 ± 3.5 |
| **Comorbidities** |  |
| Diabetes Mellitus, n (%) | 9/62 (15%) |
| Chronic Renal Failure, n (%) | 1/62 (2%) |
| Immunosuppression, n (%) | 6/62 (10%) |
| COPD, n (%) | 6/62 (10%) |
| **Primary reason for admission and ventilation** |  |
| ARDS, n (%) | 25/62 (40%) |
| Heart failure/cardiac arrest, n (%) | 2/62 (3%) |
| Septic shock, n (%) | 11/62 (18%) |
| Trauma/cerebral hemorrage/stroke, n (%) | 11/62 (18%) |
| COPD exhacerbation, n (%) | 3/62 (5%) |
| Respiratory failure, other, n (%) | 10/62 (16%) |

SOFA Sequential Organ Failure Assessment. COPD Chronic Obstructive Pulmonary Disease. ARDS Acute Respiratory Distress Syndrome.

**Figure S2. Diaphragm thickness during the first and last two days of study enrolment**

**

**

Figure E2 shows the mean change in diaphragm thickness expressed as percentage of the thickness in Day 0 from the beginning to the end of the study period.

**Figure S3. Diaphragm Thickening Fraction and clinical outcomes**

**



**

Duration of Assisted Mechanical Ventilation (Panel A) and ICU Length of Stay (Panel B) progressively increases, despite not significantly, along tertiles of diaphragmatic thickening fraction. TFdi Diaphragm Thickening Fraction. AMV Assisted Mechanical Ventilation. ICU Intensive Care Unit. LOS Lenght of stay.

**Figure S4. Correlation between Diaphragm Thickness and indices of respiratory drive and inspiratory effort**

**
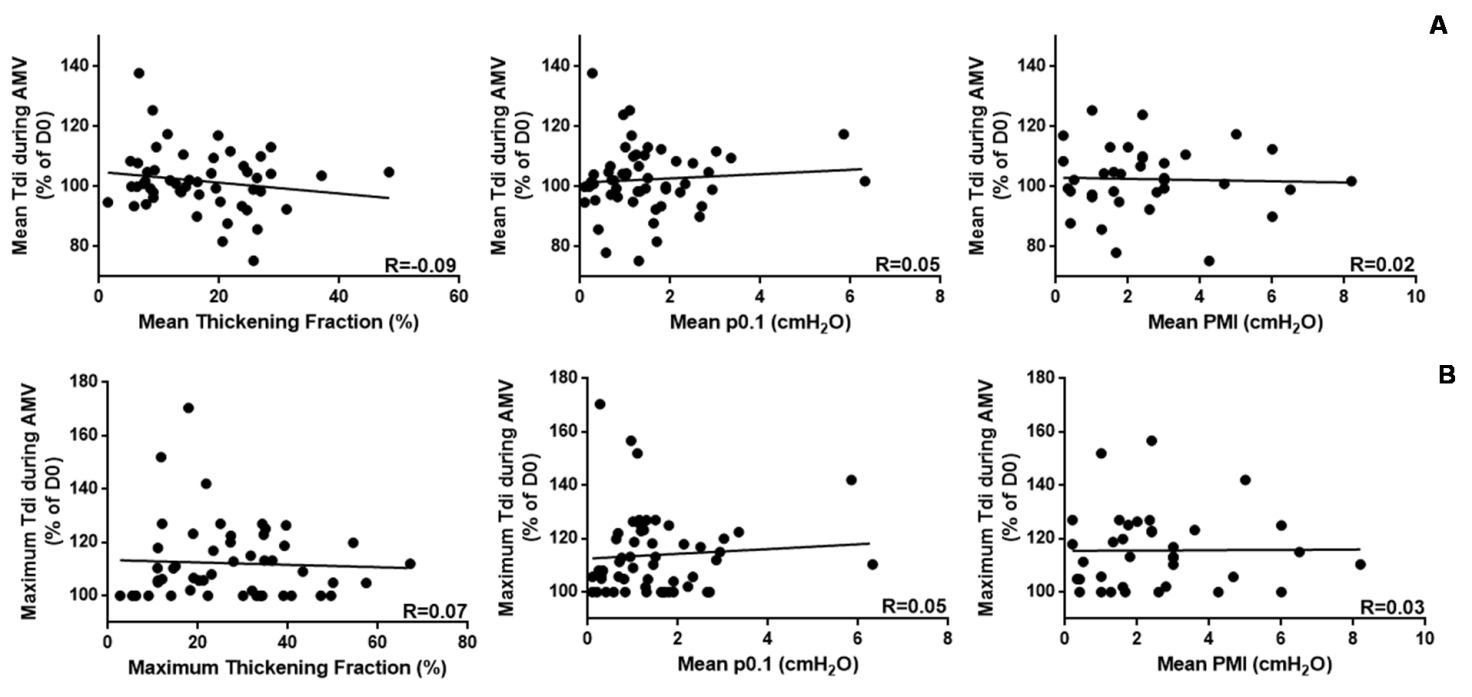
**

In Panel A, the mean diaphragm thickness during AMV period was correlated with diaphragm thickening fraction, an index of diaphragmatic activity, with p=0.1, an index of respiratory drive, and with Pressure Muscle Index, an index of respiratory effort. In Panel B, the maximum thickness reached by the diaphragm in the same study period was correlated with the same three indexes.

Tdi Diaphragm Thickness. PMI Pressure Muscle Index. D0 Day 0.
